# Spontaneous Bacterial Peritonitis: We Are Still Behind

**DOI:** 10.7759/cureus.7711

**Published:** 2020-04-17

**Authors:** Laith Numan, Ahmed Elkafrawy, Osama Kaddourah, Tim Brotherton, Lyla Saeed, Yousaf Zafar, Andrew Tomaw, John Foxworth, Leen Al-Sayyed

**Affiliations:** 1 Internal Medicine, University of Missouri-Kansas City School of Medicine, Kansas City, USA; 2 Gastroenterology and Hepatology, University of Missouri-Kansas City School of Medicine, Kansas City, USA

**Keywords:** spontaneous bacterial peritonitis, hepatology, guidelines in medicine, antibiotics, cirrhosis

## Abstract

Spontaneous bacterial peritonitis (SBP) is an infection in the ascitic fluid. Despite published guidelines, an inappropriate diagnosis of SBP is frequent. In this study, we aim to evaluate guideline adherence in diagnosing SBP. This is a retrospective study conducted between January 2015 and January 2018. Based on the American Association for the Study of Liver Diseases (AASLD) and the European Association for the Study of Liver (EASL), two authors judged guideline adherence in SBP diagnosis and management. One hundred and six patients were included in the study, and 93% were hospitalized. The mean age was 56.9 years, and 62 patients were males. In addition, Caucasians were the most common ethnicity (86.8%). The authors judged that only 52.4% of patients were appropriately diagnosed, and only 67.3% were managed with proper treatment. Inpatient mortality was documented in five patients, and the readmission rate within 30-days after discharge was 29.3%. In conclusion, SBP is a common complication of cirrhosis, which can be managed with adherence to published guidelines. In our population, guidelines were not implemented in diagnosing nearly half the SBP patients, mostly due to misdiagnosis of SBP with secondary peritonitis or non-neutrocytic bacteriascites, starting antibiotics before performing the paracentesis, and even giving broad-coverage antibiotics when not indicated. Further efforts are needed to enhance adherence to guidelines in clinical practice.

## Introduction

Liver cirrhosis is one of the common diseases in the United States, and spontaneous bacterial peritonitis (SBP) is one of the most common complications in cirrhotic patients with ascites [1]. SBP incidence in hospitalized patients with chronic liver disease and ascites varies from 10%-30%. In addition, SBP has an estimated in-hospital mortality rate of 20% [1-3]. Risk factors associated with SBP development include ascitic fluid total protein less than 1 g/dL, total serum bilirubin greater than 2.5 mg/dL, variceal hemorrhage, and a previous episode of SBP [4-7].

Misdiagnosing SBP is not uncommon, as the condition presents with vague symptoms that trigger a long list of differential diagnoses. If cirrhotic patients have ascites present with fever, abdominal pain, hepatic encephalopathy, hypotension, hypothermia, leukocytosis, or other signs and symptoms of infection, they should have a diagnostic paracentesis for ascitic fluid analysis and culture before starting antibiotics [1]. However, SBP can also be asymptomatic, so all hospitalized patients with ascites due to cirrhosis must undergo diagnostic paracentesis. Diagnostic criteria for SBP includes an ascitic fluid absolute polymorphonuclear neutrophils (PMNs) count of at least 250 cells/mm3 (0.25 x 109/L) without an intraabdominal surgically treatable source of infection [1, 8-10].

After confirming the diagnosis of SBP based on the guidelines, treatment with empiric antibiotics is warranted, even if cultures are still pending. Patients with culture-negative ascites have similar mortality rates as patients with positive cultures and, similarly, benefit from empiric antibiotic treatment [8, 11]. Empiric treatment should not begin in asymptomatic patients with PMNs <250, even if cultures grow bacteria, which could be colonization. These patients should undergo a follow-up paracentesis to differentiate between colonization from developing SBP. Conversely, if these patients are symptomatic and have signs of infection (fever, chills, abdominal pain, and hepatic encephalopathy), empiric antibiotics should be started regardless of the PMNs count [8, 11].

Starting empiric antibiotic treatment with broad-spectrum antibiotics is recommended, which can be narrowed down later once culture results are available [1, 8]. Treatment options are third-generation cephalosporins (cefotaxime and ceftriaxone), which cover most of the common bacteria causing SBP. These include *Escherichia coli, Klebsiella pneumoniae, *and *Streptococcus pneumoniae*. Cefotaxime has a reported success rate of 77-98% in treating SBP cases but is not commonly used because of limited availability in the United States [12-15]. Typically, the duration of treatment lasts five days. However, when nosocomial bacteria are suspected or previously isolated, broadening the coverage and using carbapenems or piperacillin-tazobactam is recommended [1, 2, 8].

We hypothesize that SBP is being inappropriately diagnosed. We further hypothesize that antibiotic choice and treatment duration are not accurately implemented in treating suspected SBP. In this study, we aim to evaluate adherence with guidelines in diagnosing and treating SBP. 

## Materials and methods

This is a retrospective review of all patients diagnosed with SBP in our hospital between January 2015 and January 2018. We used the diagnosis code (K65.2) to determine the population of interest. All patients were reviewed by two authors (LN and AE), and we excluded patients with missing data. The primary outcome was whether SBP was diagnosed appropriately or not. Secondary outcomes included in-hospital mortality, appropriate choice of antibiotics, and readmission rates.

Two authors (Laith Numan and Ahmed Elkafrawy) reviewed each patient and collected patients’ demographics, SBP risk factors, etiology of cirrhosis, paracentesis outcomes, ascitic fluid analysis, and cultures. Based on the American Association for the Study of Liver Diseases (AASLD) and the European Association for the Study of Liver (EASL) guidelines, two authors judged guideline adherence in SBP diagnosis and management [8, 9]. A third author resolved conflicts between the two authors. Subsequently, two authors judged if antibiotic choices were appropriate for patients correctly diagnosed with SBP.

All categorical variables were analyzed by chi-square analysis. Analysis of variance (ANOVA) and t-tests were used for continuous data, and analysis was performed using SPSS version 24.0 (IBM Inc., Armonk, US).

## Results

Baseline characteristics

One hundred and six patients were included in the study. Ninety-three percent (98/106) of them were hospitalized. The mean age was 56.9 years (range 32-87), and 62 patients were males. Caucasians were the most common ethnicity, with 86.8% (92/106). In addition, tobacco smoking was present in 41.5% (44/106), and current alcohol use was present in 28.3% (30/106) of our sample. Alcohol was the most common cause of cirrhosis accounting for 50% (53/106) of the cases, and viral hepatitis was the second, affecting 21.7% (23/106). Patient characteristics are shown in Table 1.

**Table 1:** Patients characteristics (n=106) NASH - non-alcoholic steatohepatitis

Characteristics		%	n
Age (years)	56.9 (range 32-87)		
Gender	Male	58.5%	62
Race	Caucasian	86.8%	92
	African American	2.8%	3
	Hispanic	4.7%	5
Body Mass Index (BMI) (kg/m^2^)	28.2 ± 6.9		
Smoking status	Present	41.5%	44
Alcohol use	Present	28.3%	30
Causes of cirrhosis	Alcohol	50%	53
	Viral hepatitis	21.7%	23
	NASH	12.3%	13

Primary outcomes

**Figure 1:**
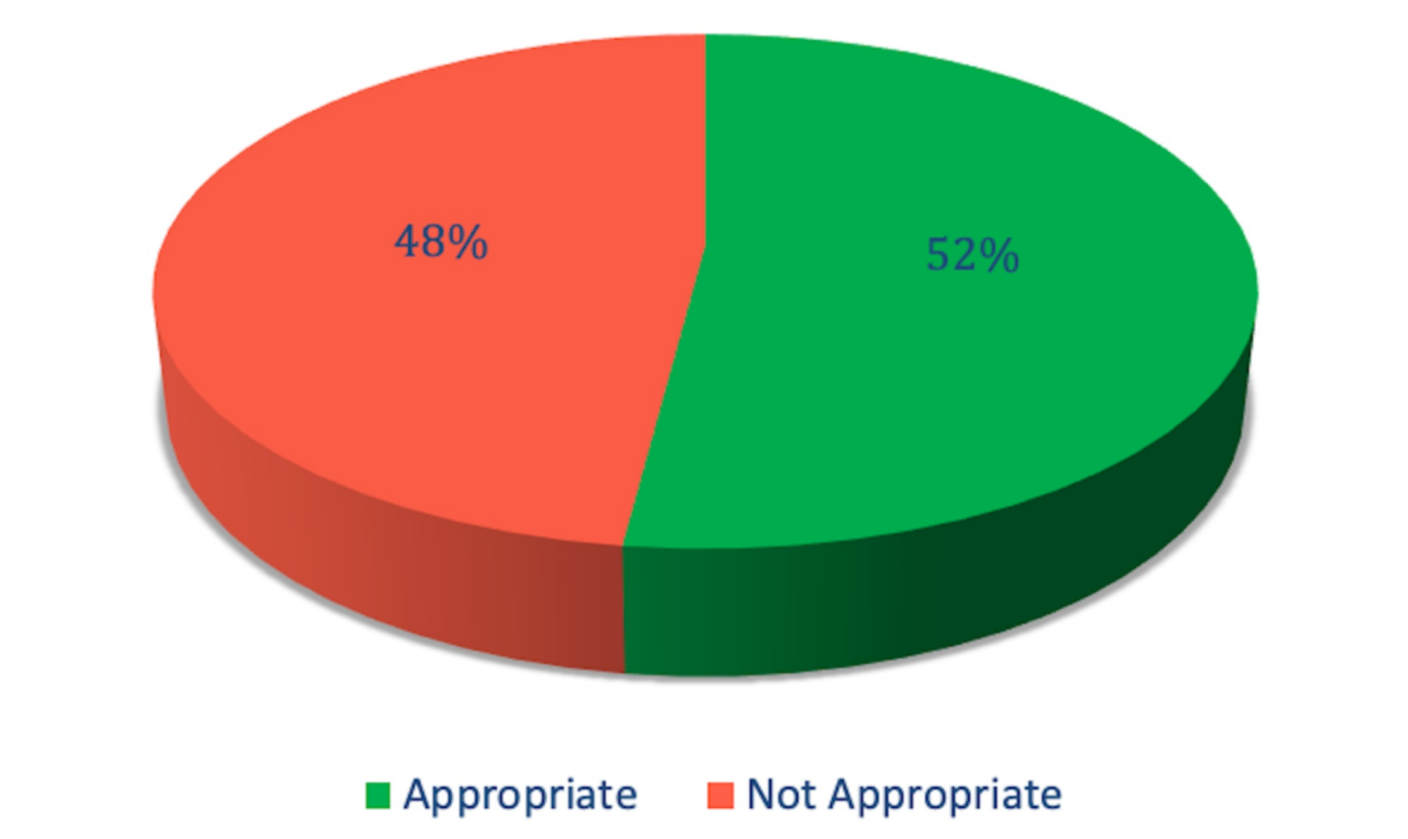
Spontaneous bacterial peritonitis diagnosis

**Figure 2:**
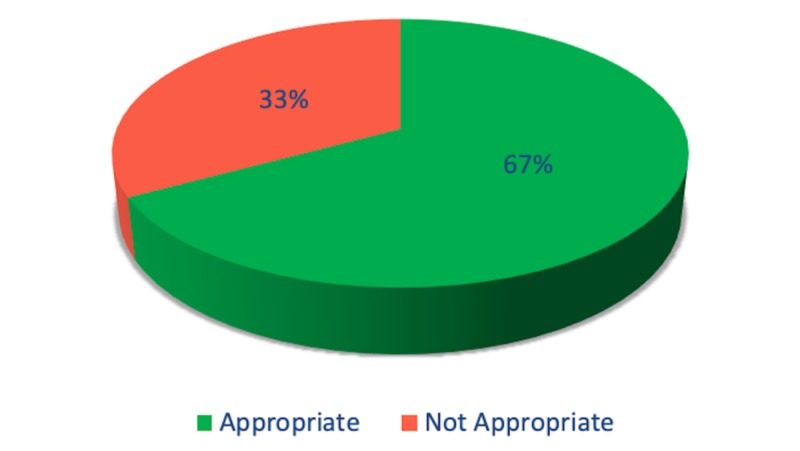
Antibiotics choice for spontaneous bacterial peritonitis

The authors judged that only 52.4% of patients were appropriately diagnosed with SBP based on guidelines (Figure 1). The remaining 47.6% patients were initially diagnosed as SBP, but later on, their symptoms were explained by other diagnoses such as urinary tract infections (43%), colitis (12%), secondary peritonitis (7%), and the rest of the patients did not have a specific diagnosis at discharge. Among the patients diagnosed with SBP, only 67.3% were managed with appropriate treatment based on recommended treatment guidelines (Figure 2). The inappropriately treated patients were either overtreated with broader antibiotics or treated with inadequate coverage. Antibiotics used in treating the subjects are shown in Table 2.

**Table 2:** Antibiotics used in appropriately diagnosed SBP SBP - spontaneous bacterial peritonitis

Antibiotics	n (%)
Aztreonam	1 (1.8%)
Cefepime	1 (1.8%)
Ceftriaxone	37 (67%)
Vancomycin	1 (1.8%)
Meropenem	2 (3.6%)
Piperacillin-tazobactam	9 (16.3%)
Metronidazole	4 (7.2%)

Secondary outcomes

Prior SBP was found in 24.5% (26/106) of patients. Mean white blood cells was 11.1 * 103 /µl (0.9-41), mean MELD-Na score was 23.5 (range 8-42), and mean albumin was 2.8 g/dl (1.6-4.9). Regarding the ascitic fluid, we found that polymorphonuclear neutrophils (PMNs) were neutrocytic (>250) in 54.8% (46/106), and 81.8% (86/106) of the ascitic cultures were negative. For the positive ascitic fluid cultures, 16.2% (17/106) grew one organism, and 1.9% (2/106) were polymicrobial. Isolated pathogens in cultures are shown in Table 3. Only one organism had multidrug resistance, and one organism was resistant to amoxicillin, the rest were pan sensitive. 

**Table 3:** Isolated pathogens in ascitic fluid cultures

Isolated pathogens in cultures	n
Escherichia coli	5
Enterococcus ssp.	5
Streptococcus spp.	5
Staphylococcus epidermidis	1
Acinetobacter baumannii	1
Staphylococcus capitis	1
Pasteurella multocida	1
Pseudomonas aeruginosa	1

Albumin was given in 62.1% (64/106) and 52.4% (54/106) on day 1 and day 3, respectively. Also, the hepatology service was consulted on 80.2% (81/106) of patients. Inpatient mortality was documented in five patients (4.9%), and 29.3% (29/106) of our subjects were readmitted within 30 days of discharge.

## Discussion

SBP is a common complication of cirrhosis and is associated with severe sequelae, including death in up to 20% of cases [1-3]. An appropriate diagnosis and correct treatment are paramount in SBP management, as untreated patient mortality is much higher and approaches 50% [16]. Guidelines regarding diagnostic criteria and appropriate treatment are well established, and improving adherence to these guidelines may improve patient outcomes. Our study investigated adherence to these guidelines for both diagnosis and treatment at our institution.

This study demonstrated that only 52.4% of patients diagnosed with SBP met appropriate diagnostic criteria. This suggests SBP was over-diagnosed in our population, with nearly half the patients not meeting diagnostic criteria. While missing a diagnosis of SBP may be more catastrophic than over-diagnosis, several patients in our study were labeled as having SBP who truly had secondary peritonitis requiring either surgical or another medical modality of treatment. Making this distinction is critical, as mortality in patients with an abdominal source of infection (e.g., a perforated bowel) who do not receive surgical intervention approaches 100% [17]. Furthermore, the mortality rate in patients with SBP who receive an unnecessary exploratory laparotomy is 80% [18]. Another cause of misdiagnosis were patients with non-neutrocytic bacteriascites, due to non-infectious bacterial colonization of the ascitic fluid, skin contamination, or even traumatic paracentesis triggering a transient bacterial leak from the bowel [19-21]. Asymptomatic non-neutrocytic bacteriascites may indicate early SBP and should be investigated further with repeat paracentesis, but if the patient is symptomatic then treatment for SBP should be initiated. Inpatient mortality was documented in five patients, which is lower than the average inpatient mortality for SBP patients. This number was probably artificially lessened by the high rate of misdiagnosis and also affected by our small sample size.

We found that out of 52.4% of patients with appropriate diagnoses, only 67.3% received appropriate treatment. Types of mistreatment included using regimens that were too broad-spectrum and treatments not providing adequate coverage for typical organisms. Inappropriate therapy with broad-spectrum agents is associated with increased cost, bacterial resistance, and antibiotic-associated complications [22, 23]. Lack of treatment or therapy with agents not covering the causal organism(s) is associated with significantly worse morbidity and mortality. Another form of mistreatment involved the initiation of antibiotics before performing paracentesis. Paracentesis should be performed before the administration of antibiotics [8]. In fact, starting antibiotics before paracentesis may have contributed to a high rate (81.8%) of our patients having negative ascitic cultures.

Our study has limitations, including being a single-center retrospective study, which might not represent other hospitals and the general population. However, we captured a diverse group of patients who might be representative of the population. Moreover, some outcome data were missing due to lack of documentation in the electronic medical records and not due to study design error, as all chart data were assessed by two independent reviewers, representing a data collection strength. We also believe some cases of SBP were missed due to incorrect coding or missed diagnosis. Regarding antibiotic choices, some confounders could have been missed, as some patients may have been started on broader antibiotics due to clinical judgment not assessable on chart review. 

This study was presented as a poster at the American College of Gastroenterology annual meeting [24].

## Conclusions

In conclusion, the guidelines regarding SBP diagnosis and treatment are evident in the literature; however, chart review of our institution’s cases showed poor adherence to guidelines. A more significant effort is necessary to apply these guidelines in clinical practice. We plan to pursue a quality improvement project to raise awareness among health care providers and hope to improve compliance with stated guidelines for both diagnosis and treatment of SBP, with an ultimate aim to improve patient outcomes.
